# Pilot to evaluate the feasibility of measuring seasonal influenza vaccine effectiveness using surveillance platforms in Central-America, 2012

**DOI:** 10.1186/s12889-015-2001-1

**Published:** 2015-07-17

**Authors:** Nathalie El Omeiri, Eduardo Azziz-Baumgartner, Wilfrido Clará, Guiselle Guzmán-Saborío, Miguel Elas, Homer Mejía, Ida Berenice Molina, Yadira De Molto, Sara Mirza, Marc-Alain Widdowson, Alba María Ropero-Álvarez

**Affiliations:** Training Programs in Epidemiology and Public Health Interventions Network (TEPHINET)/The Taskforce for Global Health, Inc., ᅟ, ᅟ; US Centers for Disease Control and Prevention (CDC), Atlanta, Georgia USA; Costa-Rican Social Security Fund (Caja Costarricense de Seguro Social), San José, Costa-Rica; Ministry of Health, San Salvador, El Salvador; Ministry of Health, ᅟTegucigalpa, Honduras; Ministry of Health, Panama City, Panama; Comprehensive Family Immunization Project, Pan American Health Organization, Washington D.C., USA; Pan American Health Organization, Ancón, Avenida Gorgas, Edificio 261, Panama City, Panama

**Keywords:** Adults, Children, Effectiveness, Hospitalization, Influenza, Vaccine

## Abstract

**Background:**

Since 2004, the uptake of seasonal influenza vaccines in Latin America and the Caribbean has markedly increased. However, vaccine effectiveness (VE) is not routinely measured in the region. We assessed the feasibility of using routine surveillance data collected by sentinel hospitals to estimate influenza VE during 2012 against laboratory-confirmed influenza hospitalizations in Costa-Rica, El Salvador, Honduras and Panama. We explored the completeness of variables needed for VE estimation.

**Methods:**

We conducted the pilot case–control study at 23 severe acute respiratory infections (SARI) surveillance hospitals. Participant inclusion criteria included children 6 months–11 years and adults ≥60 years targeted for vaccination and hospitalized for SARI during January–December 2012. We abstracted information needed to estimate target group specific VE (i.e., date of illness onset and specimen collection, preexisting medical conditions, 2012 and 2011 vaccination status and date, and pneumococcal vaccination status for children and adults) from SARI case-reports and for children ≤9 years, inquired about the number of annual vaccine doses given. A case was defined as an influenza virus positive by RT-PCR in a person with SARI, while controls were RT-PCR negative. We recruited 3 controls per case from the same age group and month of onset of symptoms.

**Results:**

We identified 1,186 SARI case-patients (342 influenza cases; 849 influenza-negative controls), of which 994 (84 %) had all the information on key variables sought. In 893 (75 %) SARI case-patients, the vaccination status field was missing in the SARI case-report forms and had to be completed using national vaccination registers (36 %), vaccination cards (30 %), or other sources (34 %). After applying exclusion criteria for VE analyses, 541 (46 %) SARI case-patients with variables necessary for the group-specific VE analyses were selected (87 cases, 236 controls among children; 64 cases, 154 controls among older adults) and were insufficient to provide precise regional estimates (39 % for children and 25 % for adults of minimum sample size needed).

**Conclusions:**

Sentinel surveillance networks in middle income countries, such as some Latin American and Caribbean countries, could provide a simple and timely platform to estimate regional influenza VE annually provided SARI forms collect all necessary information.

**Electronic supplementary material:**

The online version of this article (doi:10.1186/s12889-015-2001-1) contains supplementary material, which is available to authorized users.

## Background

Seasonal influenza causes substantive morbidity and mortality in Latin America and the Caribbean region. Children aged <5 years and adults ≥60 years with underlying medical conditions are affected most severely [1–4]. In 2003, the World Health Organization followed by the Pan American Health Organization (PAHO) and its technical advisory group on vaccine-preventable diseases in 2004, recommended vaccinating all individuals at high risk of developing severe complications from influenza virus infection. Consequently, the number of countries and territories in the Americas providing influenza vaccines through their expanded programs on immunizations (EPI) increased from 13 (29 %) in 2004 to 40 (89 %) in 2012 out of the 44 countries/territories in the region. Initially, target groups included adults aged ≥65 years, immunocompromised individuals and persons with underlying chronic conditions [5] but have since expanded to include health care workers, children (typically those aged <2 years) and pregnant women [6, 7].

Along with the increase in influenza vaccines utilization, public health practitioners have frequently explored the effectiveness of influenza vaccination in North America [8–10] but infrequently in Latin America [11–13]. Influenza vaccines are reformulated every year, and vaccine effectiveness (VE) varies between seasons, depending on the types of vaccine, their match to the circulating strains, as well as the age and health status of vaccine recipients [14]. Assessing VE may help Ministries of Health support the value of targeted influenza vaccination to prevent severe illness [15–17].

To address this evidence gap in Latin America and the Caribbean countries (LACs), we turned to severe acute respiratory infections (SARI) surveillance. Since 2007, LACs have adapted regional protocols for hospital-based SARI surveillance with laboratory diagnosis for influenza viruses [18, 19]. We aimed to evaluate if these surveillance systems could serve as platforms for annual VE estimation across the region. As a first step, we conducted a pilot in Central America to identify existing surveillance and immunization data and assess the feasibility of VE measurements. This article describes the lessons learned from this pilot in order to inform the full implementation of a VE network in other LAC countries.

The specific objectives for this pilot were to describe the variables that were routinely collected as part of SARI surveillance, those that may be used to estimate a regional adjusted VE against SARI and the data completeness of those variables. Additionally, we sought to identify data sources that would allow ascertaining vaccination status and assessed the feasibility of their use integrated to surveillance data.

## Methods

### Setting and study design

We conducted an observational case–control study at 23 SARI surveillance sentinel hospitals: 7 in Costa-Rica, 4 in El Salvador, 3 in Honduras and 9 in Panama (Table 1).

**Table 1:** Overview of influenza vaccination programs and sentinel hospitals in countries participating in the pilot influenza vaccine effectiveness case–control study in Central America, 2012

Country	Participating sentinel hospitals	Catchment populations	Vaccine introduction (public sector)	Target groups included in the pilot vaccine effectiveness case–control study	Population size for vaccination target groups	Official start date of influenza vaccination campaign (2012 influenza season)	Duration of the vaccination campaign	Influenza vaccination coverage^a^	Vaccine type and formulation used^c^	Pneumococcal vaccination among children and older adults
Costa-Rica	Secondary level (all ages): Hospital Tony Facio, Limón; Hospital Max Peralta, Cartago; Hospital San Carlos, San Carlos Alajuela; Hospital Monseñor Sanabria, Puntarenas; Hospital Escalante Pradilla, Pérez Zeledón San José; Hospital San Rafael de Alajuela, Alajuela. Tertiary level hospital (pediatric): Hospital Nacional de Niños, San José.	1,098,375 < 15 years for the pediatric hospital. 2,447,708 inhabitants for hospitals covering all ages.	2004	6 months–11 years with chronic conditions, ≥65 years.	National census projections for 2012: 365,896 < 5 years, and 316,031 ≥ 65 years.	1 February 2012	6–8 weeks	In 2013^b^, 83 % among 6–36 months, 50 % among 3–10 years and 67 % among ≥65 years.	Trivalent Inactivated virus Vaccine (TIV), Northern Hemisphere formulation.	Pneumococcal conjugate vaccine (PCV)-13 in ≤15 months.
El Salvador	Tertiary level (pediatric):Hospital del Niño Benjamín Bloom, San Salvador. Secondary level (all ages): Hospital San Juan de Dios, Santa Ana; Hospital San Juan de Dios, San Miguel; Hospital de Cojutepeque, Cojutepeque.	No catchment population data available.	2004	6–23 months, ≥60 years.	National census projections for 2012: 607,671 < 5 years, and 464,988 ≥ 65 years.	27 April 2012	6 weeks	In 2010^b^, 64 % among 6–23 months and 89 % among ≥60 years.	TIV, Southern Hemisphere formulation (changed from Northern to Southern in May 2011).	PCV-13 in <2 years and pneumococcal polysaccharide vaccine (PPV)-23 in ≥60 years.
Honduras	Tertiary level (all ages): Instituto Hondureño de Seguridad Social, San Pedro Sula; Hospital Catarino Rivas, San Pedro Sula; Instituto Cardiopulmonar “TORAX”, Tegucigalpa. Secondary level: Hospital Militar, Tegucigalpa.	No catchment population data available.	2003	6–35 months with chronic conditions, ≥60 years.	National census projections for 2012: 1,085,293 < 5 years, and 358,553 ≥ 65 years.	15 November 2011	6 weeks	In 2011^b^, 71 % among children with chronic conditions. In 2012 73 % among ≥65 years.	TIV, Northern Hemisphere formulation.	PCV-13 in <1 year (as per Expanded Programme on Immunization schedule). In 2011–2012, PPV-23 in individuals 2–59 years with chronic conditions and ≥60 years (vaccine donation).
Panama	Tertiary level (pediatric): Hospital del niño, Panama City; Hospital de Especialidades Pediátricas, Panama City; Hospital José D. De Obaldía, Chiriquí. Secondary level (all ages): Hospital José Luis “Chicho” Fábrega, Veraguas; Hospital Rafael Hernández, Chiriquí; Hospital Rafael Estévez, Coclé; Hospital Joaquín Pablo Franco, Los Santos.	No catchment population data available.	2005	6–59 months, ≥60 years.	National census projections for 2012: 3,787,511 < 5 years, and 384,754 ≥ 65 years.	15 April 2012	Vaccination concentrated during the “vaccination week of the Americas” (last week of April) and offered throughout the season depending on stocks availability and expiration dates.	In 2012, 69 % among 6–59 months and 83 % among ≥60 years.	TIV, Southern Hemisphere formulation.	PCV-13 in <1 year, and PPV-23 in ≥60 years.

^a^As officially reported by the Expanded Programs on Immunization

^b^Vaccination coverage estimates unavailable for 2012

^c^Northern and Southern formulations were identical in 2012 including an: A/California/7/2009 (H1N1)-like virus, A/Perth/16/2009 (H3N2)-like virus and B/Brisbane/60/2008-like virus

Five were pediatric hospitals and 17 covered all ages. Catchment population were unavailable except for Costa-Rica (1,098,375 < 15 years for the national pediatric hospital and 2,447,708 inhabitants for hospitals covering all ages). Population census data for 2012 suggest a total target population at risk of influenza across all countries of 5,846,371 children ≤ 5 years and 1,524,326 adults ≥65 years (Table 1.).

Surveillance staff at participating hospitals identified, as part of routine surveillance, patients with SARI defined as temperature >38 °C or history of fever, cough, difficulty breathing, and hospitalization. A case was defined as an influenza virus positive by RT-PCR in a person with SARI. A control was a RT-PCR negative for influenza in a person with SARI. Depending on the hospital, surveillance nurses, medical doctors or hospital epidemiologists collected respiratory specimens (i.e., nose and throat swabs, nasal washes or aspirates) from SARI case-patients. Hospitals aimed to collect specimens from all SARI case-patients in Costa-Rica and Honduras and from a convenience sample of 5 weekly SARI case-patients in El Salvador and Panama as per surveillance protocols. Specimens were tested for influenza viruses through reverse transcription polymerase chain reaction (RT-PCR) following the CDC protocol for detection and typing/subtyping of influenza viruses [20]. We selected 3 controls per case, frequency matching by age-group (children aged 6 months–11 years and adults aged ≥60 years) and matching by week of symptoms onset ± 2 weeks (when controls were unavailable from the same week, we selected controls in patients with symptom onset ±2 weeks from that of case-patients).

### Participants

The population under study consisted of children and older adults targeted for government-sponsored influenza vaccination: children 6–59 months (El Salvador and Panama); 6–23 months with preexisting conditions (Honduras); 6 months–11 years with preexisting conditions (Costa-Rica) and adults ≥60 years (El Salvador, Honduras and Panama) and ≥65 years (Costa-Rica). Thus participants were persons belonging to these target groups, seeking care at any of the participating hospitals during 2012, with a specimen collected with ≤10 days since the onset of symptoms and no contra-indication for influenza vaccines. Only vaccination through the EPI was considered in this vaccination program evaluation that covers the majority of influenza vaccinations among children and older adults in Central America. EPI estimates of coverage for seasonal influenza vaccine ranged from 64 to 83 % among young children and 67 to 89 % among older adults (Table 1).

### Variables

We reviewed SARI case-report forms from the 4 participating countries in order to identify routinely collected information that could be used for VE estimation [21]. Key variables were defined as sex, age, date of onset of symptoms, date of respiratory specimen collection, influenza virus RT-PCR results, presence or absence of at least one preexisting condition, and influenza vaccination status and date in the current season. Preexisting conditions were defined as asthma, cystic fibrosis, chronic pulmonary disease, obesity, diabetes, immunosuppression, immunodeficiency or heart disease in Costa-Rica; congenital malformations, immunosuppression, chronic diseases, or neurological disease in El Salvador; heart disease, chronic pulmonary disease, diabetes, cancer, immunosuppression, chronic alcoholism, obesity or other conditions in Honduras; and chronic diseases or immunosuppression in Panama. Additionally, we collected the influenza vaccination status in the prior season, and pneumococcal vaccination status to explore confounding/effect modification. Although SARI case-report forms also included variables on antiviral use and its corresponding date of administration, participating countries chose not to compile this information for the pilot because antivirals are infrequently used in Central America [22].

### Data sources/measurement

We developed a protocol drawing on experience from sentinel surveillance-based VE studies in the United States, Canada, Australia, and Europe [21, 23–26]. The primary data sources used to fill SARI case-report forms were typically medical records or physicians’ interviews for demographic and clinical data, and vaccination cards or medical records for vaccination status. Surveillance staffs liaised with reference laboratories to obtain influenza virus RT-PCR results. The dates of respiratory specimen collection were recorded and provided by the person collecting the specimen. Preexisting conditions were either documented in medical records or self-reported by patients during the medical consultation. Information was compiled mostly from paper reviews and entered into an excel spreadsheet by surveillance staff. National teams reviewed reports of SARI case-patients with onset of symptoms during 2012 and their RT-PCR results.

As part of routine SARI surveillance, hospital staff collected the influenza vaccination status (vaccinated/unvaccinated), the total number of vaccine doses and the date of the last dose received. This information was typically retrieved from vaccination cards brought in by the patients upon hospitalization or from medical records. For the purpose of the evaluation, we encouraged surveillance staff to obtain vaccination cards during hospitalization or liaise with EPI local or regional teams to obtain information from vaccination registers or other EPI records when necessary. In the latter case, the patient’s name, date of birth, and residence details were matched to EPI data sources. If unavailable, the EPI staff contacted patients by telephone or visited households to review vaccination cards. Patients/parents were asked to provide exact dates of vaccination and vaccination centers so that EPI staff can verify the information. Note that EPI staff had no access to the influenza status of SARI patients or to other clinical information.

We defined exposure as vaccination with the locally available trivalent inactivated influenza vaccine during 2012 with the Southern Hemisphere formulation for Costa-Rica, El Salvador and Panama; and during the November-December 2011 vaccination campaign in Honduras using the Northern hemisphere formulation. An individual was considered vaccinated if he/she received the vaccine at least 14 days before the onset of SARI symptoms [27]. We considered a child aged ≤9 years fully vaccinated if he/she received two doses of vaccine as recommended by WHO [28] and partially vaccinated if he/she received one dose. We considered a person vaccinated against pneumococcal disease if he/she had an up to date vaccination record according to local recommendations as determined by EPI staff.

### Bias

We reviewed published reports from VE studies using surveillance-based test-negative designs in order to identify potential confounders and selected those for which data was collected as part of routine surveillance [21, 23–26]. These factors included the age, sex, date of symptoms onset as a proxy for calendar time, presence of at least one preexisting condition, receipt of pneumococcal vaccines (as a proxy for access to EPI vaccines and of influenza vaccine in the previous season among older adults). The effect of these variables would be examined in stratified analysis and by inclusion/exclusion in logistic regression models. Selected variables would be controlled for in final models providing adjusted VE. In order to avoid misclassification of the outcome, we collected data to calculate the number of days between symptoms onset and specimen collection and exclude SARI case-patients with >10 days between them from the analysis.

### Study size

Using a formula for unmatched case–control studies with 3 controls per case, we calculated the minimum number of SARI case-patients per target group that we would need to detect an odds ratio (odds of vaccination among cases/odds of vaccination among controls) significantly different from 1. We would need at least 138 influenza cases and 414 controls per age-group at the regional level, to detect a hypothesized odds ratio of 0.5 (i.e., VE of 50 %), if 30 % of controls were vaccinated [unpublished data, Costa-Rica 2011]. We used 80 % power, and an alpha-type error of 5 % [29, 30]. Assuming that ~16 % of SARI case-patients would test positive for influenza in the 4 countries [unpublished 2011 surveillance data; 31], we sought to identify ≥837 SARI case-patients per target group with all necessary information to reach sample size.

### Statistical methods

We calculated the proportion of SARI case-patients with information about all variables sought to estimate adjusted VE. We described data sources used for influenza vaccination status ascertainment. To determine the sample size for a potential VE analysis, we restricted the sample to SARI case-patients with an onset of symptoms 15 days after the official start of influenza vaccination in each country. We also excluded SARI case-patients with onset of symptoms preceding the first laboratory-confirmed influenza case or occurring 2 weeks after the last laboratory-confirmed influenza case in each country. To avoid misclassification, we excluded SARI case-patients with >10 days between symptoms onset and specimen collection (if this exclusion criterion was not applied by the country) and those for whom this information was unavailable (i.e., missing date of symptoms onset or of sample collection).

### Ethical considerations

The ethics committees of the Costa Rican Social Insurance Fund and of participating hospitals in Costa-Rica approved the protocol. The Ministries of Health in El Salvador, Honduras, Panama, and the US CDC waived its review because it was considered a program evaluation using surveillance data. We did not collect personal identifiers. Data was anonymized at the country level by assigning alpha-numeric codes to subjects. Data was securely stored electronically at the Ministry of Health in El Salvador, Honduras and Panama and at the Costa-Rican Social Insurance Fund in Costa-Rica).

## Results

### SARI case-patients identified

During 2012, 1,186 SARI case-patients were hospitalized: 647 (55 %) in Costa-Rica, 334 (28 %) in El Salvador, 107 (9 %) in Honduras and 98 (8 %) in Panama. Seven hundred and seventy-seven (66 %) were children aged 6 months–11 years (735 [62 %] <5 years old), and 409 (34 %) were adults aged ≥60 years. Half of reported SARI case-patients were male (603 [51 %]).

**Fig. 1:**
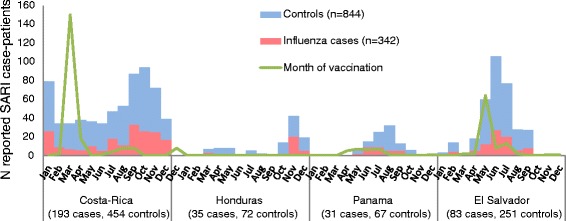
Distribution of severe acute respiratory infections (SARI) case-patients reported by month of onset of illness and month of vaccination, pilot influenza vaccine effectiveness case–control study in Central-America, 2012 (*N* = 1,186)

Of 1,186 SARI case-patients, 342 (29 %) tested positive for an influenza virus and were designated as cases and 844 (71 %) tested negative for an influenza virus and were classified as controls: 212 cases and 565 controls were children and 130 cases and 279 controls were older adults. Influenza cases peaked during June-July in Costa-Rica, El Salvador and Panama and during September-November in Costa Rica and Honduras (Fig. 1) (Additional file 1).

### Completeness of surveillance data

All 1,186 SARI case-patients had information on age, gender, and the date of onset of symptoms. The date of respiratory specimen collection was available for 1,127 patients (95 %) and 338 (29 %) lacked information about the presence or absence of preexisting conditions (Table 2).

**Table 2:** Proportion of identified severe acute respiratory infections case-patients with complete information for selected variables, pilot case–control study for influenza vaccine effectiveness in Central-America, 2012 (*n* = 1,186)

	6 months − 11 years (*n* = 777)		≥60 years (*n* = 409)	
	Influenza cases	Controls	Influenza cases	Controls
	*n* = 212	*n* = 565	*n* = 130	*n* = 279
Age	100 %	100 %	100 %	100 %
Gender	100 %	100 %	100 %	100 %
Clinical information				
Date of onset of illness	100 %	100 %	100 %	100 %
Date of specimen collection	92 %	95 %	95 %	100 %
Preexisting conditions (yes/no)	67 %	61 %	88 %	87 %
Vaccination information				
Vaccination status for current influenza vaccine^a^	88 %	88 %	82 %	91 %
Date of current influenza vaccine receipt	100 %	100 %	100 %	100 %
Vaccination status for a second annual dose among children 6 months–9 years^b^	67 % (121/193)	57 % (289/503)	NA^c^	NA
Prior season influenza vaccination	96 %	96 %	84 %	94 %
Pneumococcal vaccination status^d^	80 %	86 %	34 %	19 %
Number of complete records for key variables for vaccine effectiveness analyses^e^	170 (80 %)	471 (83 %)	100 (77 %)	253 (91 %)
Number of complete records for all variables collected^f^	151 (71 %)	458 (81 %)	34 (26 %)	51 (18 %)

^a^For receipt of at least one dose among children and older adults and after active/enhanced vaccination status ascertainment

^b^WHO recommends 2 doses among children 6 months–­9 years vaccinated for the first time

^c^NA = Not applicable

^d^Vaccination up-to-date (yes/no) according to local recommendations

^e^Defined as age, gender, dates of onset of symptoms and specimen collection, current vaccine status and date, presence of at least one preexisting condition, and country

^f^Key variables, pneumococcal vaccination and prior influenza vaccination

### Vaccination status ascertainment

One quarter (293) of SARI case-patients had 2012 vaccine status originally recorded in their SARI case-report forms: 234 (30 %) of 777 children and 59 (14 %) of 409 older adults. We did not obtain information on the original completeness of the SARI case-report forms for the 2011 vaccination. No distinction could be made between first and second doses in potentially vaccine-naïve children in SARI case-report forms (i.e., children ≤ 9 years, unvaccinated or with no information about prior influenza vaccination). Electronic nominal vaccination registers were available in Costa-Rica nationally and in Panama for 80 % of health facilities but not in Honduras and El Salvador. After seeking vaccination history from EPI registers, vaccination cards, medical records, and other sources (Table 3), 88 % (1,042) of SARI case-patients had 2012 and 94 % (817) had the prior season vaccine status information (2011 vaccine for Costa-Rica, El Salvador and Panama; November–December 2010 campaign for Honduras). All vaccinated individuals had available vaccination dates. Out of 685 children aged ≤11 years and previously unvaccinated or with missing information about prior vaccination, 398 (57 %) had information about the receipt of a second influenza vaccine dose. Restricting to 132 children that had additionally reported being vaccinated with at least one dose, 59 (45 %) had information on a second dose. Pneumococcal vaccination status was available for 754 patients (64 %).

**Table 3:** Description of data sources used for ascertaining vaccination status in severe acute respiratory infections case-patients identified, pilot case–control study for influenza vaccine effectiveness in Central-America, 2012 (*n* = 1,186)

	Costa-Rica (*n* = 647)		El Salvador (*n* = 334)		Honduras (*n* = 107)		Panama (*n* = 98)	
Data source	6 months − 11 years with chronic conditions, n = 339 (%)	≥65 years, *n* = 308 (%)	6 − 59 months, *n* = 287 (%)	≥60 years, *n* = 47 (%)	6 − 35 months, *n* = 60 (%)	≥60 years, *n* = 47 (%)	6 − 59 months, *n* = 91 (%)	≥60 years, *n* = 7 (%)
Surveillance forms or database^a^	184 (54)^a^	39 (13)^a^	20 (7)	7 (15)	13 (22)^a^	13 (28)^a^	17 (19)^a^	0 (0)
Vaccination cards	184 (54)	39 (13)	60 (21)	0 (0)	44 (73)	17 (36)	17 (19)	0 (0)
Nominal vaccination registers	150 (44)	262 (87)	0 (0)	0 (0)	0 (0)	0 (0)	19 (21)	0 (0)
Local EPI records or vaccination facilities records	– ^b^	– ^b^	29 (0)	2 (4)	2 (3)	17 (36)	0 (0)	0 (0)
Verbal report of vaccination card review (over the phone)	5 (2)	– ^b^	0 (0)	0 (0)	13 (22)	13 (28)	0 (0)	5 (71)
Medical records	0 (0)	– ^b^	15 (5)	8 (17)	0 (0)	0 (0)	52 (57)	0 (0)
Unspecified document reviewed^c^	0 (0)	0 (0)	110 (38)	25 (53)	0 (0)	0 (0)	0 (0)	0 (0)
Unreachable patient/undocumented	0 (0)	7 (0)	53 (18)	5 (11)	1 (2)	0 (0)	3 (3)	2 (29)

^a^Surveillance forms information was based on the review of vaccination cards in Costa-Rica and Panama, and on over-the-phone readings of vaccination cards in Honduras

^b^Data source not used

^c^May include vaccination card or any other paper document

### Completeness of VE case–control study data

After completing the review of vaccination information, 694 of 1,186 SARI case-patients (59 %) had information on all variables collected including potential confounders and 994 (84 %) had information on variables selected *a priori* for VE analyses: 82 % of children (641/777) and 86 % of adults (353/409).

### Proportion of vaccinated SARI case-patients

Out of 1,042 SARI case-patients with available vaccination history, 320 (31 %) had received at least one dose of the 2012 influenza vaccine. Nineteen (6 %) received the vaccine <2 weeks before illness onset and 55 (17 %) after the illness onset.

Among 716 children aged 6 months–11 years, 151 (22 %) had received at least one dose of influenza vaccine in 2012. Of 147 vaccinated children that also had information about prior season vaccine, 18 (12 %) were also vaccinated in 2011. Among 56 children aged 6 months–11 years with no prior vaccination, information about a second dose and who reported having received at least one dose of vaccine in 2012, 9 (16 %) had received 2 doses and 47 only one dose. Forty-seven percent of adults aged ≥60 years (169/361) received the vaccine in 2012. Of 292 adults vaccinated in 2012 that had information about the prior season vaccine; 35 (12 %) were previously vaccinated in 2011.

### SARI case-patients included in the analysis

**Fig. 2:**
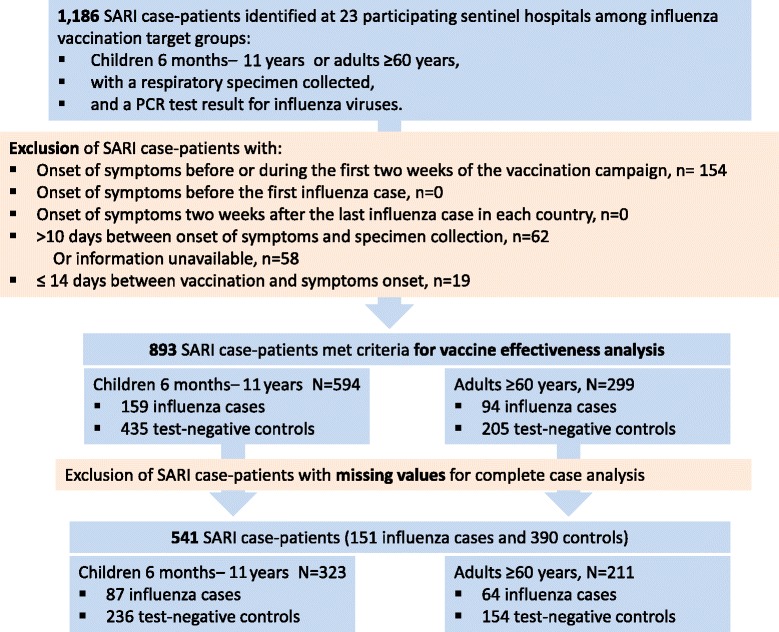
Selection of severe acute respiratory infections (SARI) case-patients for vaccine effectiveness analysis, pilot influenza vaccine effectiveness case–control study in Central America, 2012

SARI case-patients received influenza vaccine from 10 months before the onset of symptoms to 9 months after the illness onset (median of 63 days between vaccination and symptoms onset [~2 months], interquartile range = 4.5 months) (Fig. 1). Out of 1,186 SARI case-patients identified, we excluded 154 (13 %) patients that had initiated illness before or within the first 2 weeks of vaccination campaigns and 19 (1.6 %) with <2 weeks between vaccination and symptoms onset. We also excluded 62 (5.2 %) with samples collected >10 days after symptoms onset, 58 (4.9 %) with information missing on the number of days between symptoms onset and sample collection (Fig. 2). Thus, we selected 253 cases and 640 controls that met the VE case–control study criteria. We further excluded 126 patients that had no information on vaccination status and 226 that lacked information on preexisting conditions. Thus, 87 cases and 236 controls aged 6 months–11 years and 64 cases and 154 controls aged ≥60 years were eligible for complete case VE analysis. The sample size for a regional VE estimate for children lacked 37 % of the minimum number of cases and 43 % of controls, and adults lacked 43 % of cases and 63 % of controls to meet our minimum sample size to estimate adjusted VE.

## Discussion

Our findings from a pilot case–control study for estimating regional influenza VE conducted in 4 Central American countries suggest that it is feasible to use the current SARI surveillance platforms (variables, processes and infrastructure) to measure a target group-specific adjusted VE with minor adjustments to data collection and through integration with EPI data. The sustainability of annual measurements of VE will depend largely on countries’ efforts to improve the completeness of the vaccination variables in SARI case-report forms or in electronic immunization registers.

### Feasibility of sentinel platforms-based influenza VE evaluation

SARI surveillance gathered information on variables about the outcome, exposure, and potential confounders or effect modifiers of influenza VE. In Central America, the completeness of information on demographic and clinical characteristics was generally high. To better assess exposure to influenza vaccines, the number of doses among potentially vaccine-naïve children, and their corresponding dates of receipt would need to be included in SARI case-report forms. Since this pilot, PAHO has updated its regional SARI surveillance guidelines to include these variables in the SARI case-report forms.

Historically vaccination history has not been a mandatory variable to ascertain during routine surveillance data collection. Nevertheless, we found that it was possible to complete vaccination history by encouraging surveillance staff to review vaccination cards during hospitalization or by reviewing other EPI data sources. Pneumococcal vaccination status information among older adults remained poor (24 %), however, probably because this vaccine is in the EPI schedule of only 2 countries.

Ascertaining vaccination status was most efficiently done using nominal vaccination registers. These registers were particularly valuable for older adults who, unlike the parents of young children, infrequently carry their vaccination cards. Although nominal registers are uncommon in Latin America, many countries are currently in the process of developing or implementing them. The availability of such registers may render VE evaluations less costly and time consuming in the future.

### Recommendations

A key lesson learned from this pilot was the importance of integrating work between influenza surveillance, reference laboratories and the EPI to meet the vaccination program evaluation objectives. Therefore, as part of the project implementation in 2013, we officially established multi-disciplinary/multi-institutional teams and clearly defined their roles and responsibilities. Surveillance staff in the region often has a high turnover and organizing in-country trainings prior to the influenza season may contribute to improvements in data collection for VE estimation while benefitting surveillance in general. We recommend that such trainings emphasize the importance of collecting quality surveillance data with the review of vaccination documents/cards during hospitalization in order to reduce misclassification and the risk of potential bias. Moreover, surveillance staff should differentiate between an unvaccinated individual and one with no available vaccination information and understand the purpose of collecting information about covariates such as prior influenza or pneumococcal vaccination.

While surveillance forms were quite similar in their formulation of variables, data collection tools used to share countries’ data for the regional analysis were sub-optimal. Open-end Excel databases were difficult to clean as variables coding was only standardized for 2 countries. Thus, in preparation for the implementation phase of our vaccination program evaluation, we developed a web-based closed-ended online questionnaire for countries that enter paper SARI case-reports data and an online module allowing for the upload of sub-databases from electronic surveillance systems, using a common codebook.

### Timing of vaccination

Our data confirmed that vaccination typically took place before the occurrence of laboratory-confirmed influenza hospitalizations in El Salvador, Panama and Costa-Rica. In the case of Honduras, the mid-year incidence of influenza cases may be underrepresented in this dataset that reports a higher number of influenza cases at the end of the year, possibly due to a surge in the recruitment of influenza surveillance staff in October. Contrary to countries from temperate areas of the Americas, it has been challenging for countries of the American Tropics such as Central America, to define the seasonality of influenza epidemics to define the best timing and vaccines formulation to use. Nevertheless, in recent years these countries have made substantial progress in collecting epidemiological and virological data that have allowed countries such as Honduras and Costa-Rica to adjust their vaccination policies opting for the Southern Hemisphere formulation and vaccination in April-May [Durand et al. submitted manuscript].

### Increasing sample size

We could not reach the minimum sample size needed for VE calculations with the number of sentinel hospitals included in this pilot. We collected data from 4 small Central American countries where vaccination coverage was low and RT-PCR confirmed influenza hospitalization was a rare outcome. Indeed, 22 % of hospitalized children and 47 % of adults had received at least one dose of influenza vaccine, both much lower than the official vaccine coverage estimates. This may be partly explained by the expected differences between hospitalized populations and the general population targeted for vaccination, but also by the difficulty in estimating true denominators when measuring vaccine coverage using the administrative method which divides the number of doses administered by the size of the target population [32].

First, we identified an insufficient number of adults required for the adjusted VE estimates (409/837; 49 % of target adult SARI case-patients versus 92 % for children). Then we lost 46 % of children (271/594) and 29 % of adults (88/299) selected for the analyses due to missing variables required for adjusted VE estimates. Very wide confidence intervals, suggesting that true VE lies within an extremely large range, are of little value for public health decision-making. Losses in sample size could be reduced by strengthening data collection during surveillance and increasing the number of sentinel hospitals across LAC countries especially among countries with higher influenza vaccine coverage.

### Next steps and perspectives

In February 2013, PAHO, CDC and TEPHINET launched the network for influenza vaccine evaluations in Latin America and the Caribbean known as REVELAC-i for its acronym in Spanish (*Red para la Evaluación de Vacunas En Latino América y el Caribe–influenza*) that would allow more countries to participate and facilitate more powerful analyses. The aim of the network is to facilitate the collection and sharing of high quality data between influenza surveillance and EPIs in order to estimate VE and impact. As of March 2015, 15 countries have joined the network (Argentina, Brazil, Chile, Colombia, Costa-Rica, Cuba, Ecuador, El Salvador, Honduras, Mexico, Nicaragua, Panama, Paraguay, Peru and Uruguay); 10 of which have collected, analyzed, and shared data during 2013.

Unlike for other vaccines, evaluating the impact of an influenza vaccination programs would require several years of VE, disease burden, vaccine coverage, and population denominator data to account for the variability between the influenza seasons. Consequently, the setup of annual monitoring of VE is important for LACs. Moreover, VE data from LACs may inform the “Global Initiative for Vaccine effectiveness” that compiles VE data bi-annually for the WHO “Meeting on the composition of influenza vaccines”, contributing to the body of evidence for the Southern Hemisphere for which periodic reporting is currently mostly done by Australia and New Zealand. This contribution is also in line with the reporting of events or critical findings of concern, identified through the evaluation of active pharmaceutical products under the Annex 2 of WHO’s International Health Regulations [33].

Findings regarding surveillance infrastructure and field data may positively support national efforts towards better and timelier influenza surveillance and response to influenza epidemics.

## Conclusion

Sentinel SARI surveillance networks in middle income countries such as those participating in REVELAC-i and SARInet in the Americas could annually estimate influenza VE with minor adjustments to their current surveillance practices, provided that these networks generate quality data (e.g., influenza vaccination history). In future influenza seasons, REVELAC-i will aim to aggregate data from more countries with robust surveillance systems and immunization records.

## Additional file

Additional file 1: Figure S1B. Distribution of severe acute respiratory infections (SARI) case-patients in Central-America, pilot influenza vaccine effectiveness evaluation, 2012 (*N* = 1,186).
