# Supplementary material for: Pilot to evaluate the feasibility of measuring seasonal influenza vaccine effectiveness using surveillance platforms in Central-America, 2012
Source: BMC Public Health. 2015 Jul 17;15:673. doi: 10.1186/s12889-015-2001-1 (PMC4504410; doi:10.1186/s12889-015-2001-1)

Additional file 1: FIGURE S1B. Distribution of severe acute respiratory infections (SARI) case-patients in Central-America, pilot influenza vaccine effectiveness evaluation, 2012 (N=1,186).


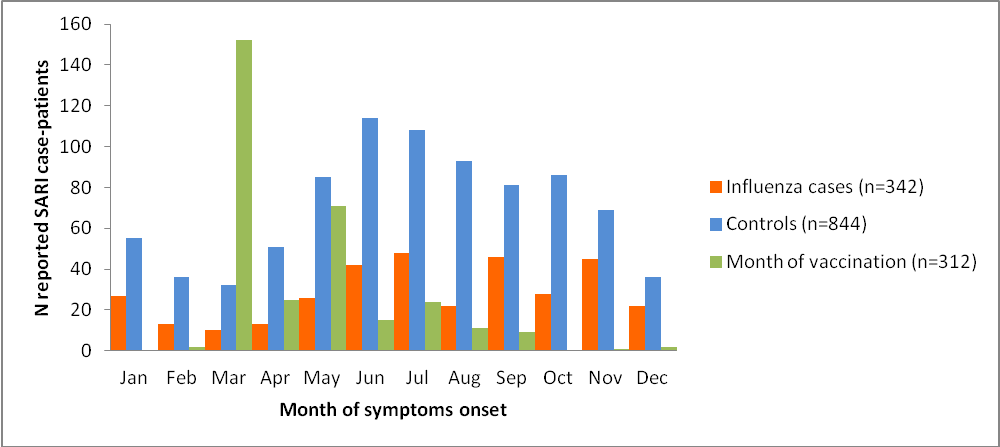

Supplement: Additional file 1: Figure S1B. — Distribution of severe acute respiratory infections (SARI) case-patients in Central-America, pilot influenza vaccine effectiveness evaluation, 2012 (N = 1,186). [file 12889_2015_2001_MOESM1_ESM.doc]
